# Necrotizing Fasciitis Masquerading as Stroke: A Diagnostic Dilemma

**DOI:** 10.7759/cureus.60870

**Published:** 2024-05-22

**Authors:** Taylor Locklear, Alexander B Holland, Saptarshi Biswas

**Affiliations:** 1 Surgery, Grand Strand Medical Center, Myrtle Beach, USA; 2 Medicine, Edward Via College of Osteopathic Medicine, Spartanburg, USA

**Keywords:** necrotizing fasciitis management, emergent general surgery, cecal perforation, acute care surgery and trauma, abdominal wall necrotizing fasciitis

## Abstract

Necrotizing fasciitis (NF) poses a diagnostic challenge due to its rarity and similarity in presentation with other critical conditions. We report a case of a 79-year-old male who initially presented with altered mental status and stroke-like symptoms; he was ultimately diagnosed with abdominal wall NF spreading to the lower extremity. Despite a history of cecal mass perforation noted in prior imaging, the patient had been discharged from an outside emergency room on antibiotics, highlighting a missed opportunity for early intervention. Subsequent deterioration led to sepsis, organ failure, and ultimately, the detection of NF. Prompt recognition of subtle skin changes and laboratory abnormalities, along with a detailed physical examination, is essential for a timely and accurate diagnosis. Surgical debridement, coupled with broad-spectrum antibiotics, remains the cornerstone of treatment. Delays in surgical management significantly increase mortality, emphasizing the importance of prompt diagnosis and intervention. This case underscores the necessity for heightened awareness among healthcare providers to recognize NF promptly, especially when its clinical presentation overlaps with other critical conditions. Multidisciplinary collaboration and continued education are imperative to improve outcomes and prevent delays in the diagnosis and treatment of NF.

## Introduction

Necrotizing fasciitis (NF) is a rare, rapidly progressing infection of the subcutaneous tissues and fascia of the skin, with roughly 1000 new cases reported in the United States annually [1]. The average mortality rate of NF is about 20%, even with optimal treatment [2]. It most commonly affects the abdomen, perineum, and lower extremities, although it can occur in any part of the body [1,3]. Given the rapid progression and significant morbidity and mortality associated with the disease, prompt recognition and surgical management is critical. However, roughly 15% of NF cases are admitted to the hospital with an incorrect diagnosis [2]. We discuss a case of abdominal wall NF spreading to the lower extremity in an adult patient presenting with altered mental status and stroke-like symptoms.

## Case presentation

A 79-year-old male with a medical history of type II diabetes mellitus, prostate cancer (in remission), hyperlipidemia, and hypertension initially presented to the emergency department with altered mental status and concern for a stroke. On arrival, he was noted to be lethargic, unresponsive to commands, and aphasic. His National Institutes of Health (NIH) stroke scale score was 27. He also had a left-eye gaze deviation. The patient’s wife, who arrived shortly after the patient’s presentation, mentioned that the patient had exhibited gait instability, frequent urination with an inability to control his bladder, and abdominal pain over the last few days. She also mentioned that the patient had a fall the previous night, which had prompted her to take him to an outside emergency department. Per the discharge paperwork, CT of the patient’s abdomen and pelvis showed evidence of colitis with a perforated cecal mass. The patient had been sent home with oral antibiotics, though the wife stated that his confusion and fatigue had continued to worsen after discharge.

**Figure 1:**
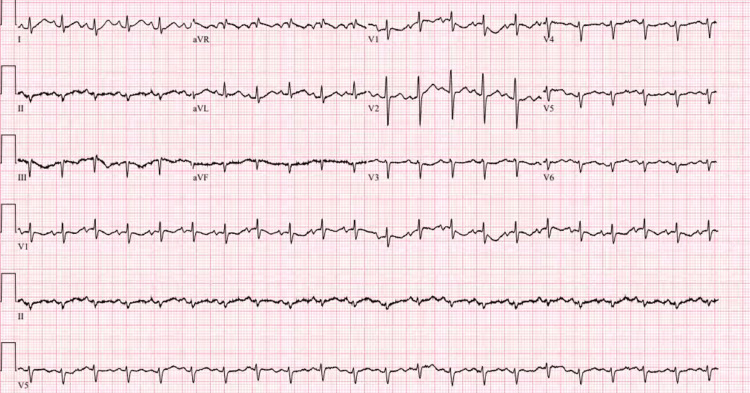
Electrocardiogram showing sinus tachycardia

The vital signs on presentation to the emergency department were significant for fever of 101 °C, blood pressure of 188/134 mmHg, oxygen saturation of 98% on two liters via nasal cannula, and tachycardia into the 130s. The EKG showed sinus tachycardia (Figure 1). Lab workup revealed a leukocytosis of 14.6 K/mm^3^ with CO_2_ of 19 mmol/L, and lactic acid of 5.8 mmol/L, indicating sepsis. The anion gap was 15 mEq/L. The creatinine was also elevated at 2.4 mg/dL, along with a total bilirubin of 3.9 mg/dL, aspartate aminotransferase (AST) of 228 units/L, alanine transaminase (ALT) of 80 units/L, and prothrombin time (PT) of 20 seconds with an international normalized ratio (INR) of 1.75 (Table 1).

**Table 1:** Pertinent laboratory values of the patient on presentation to the emergency department WBC: white blood cells; CO_2_: carbon dioxide; Crt: creatinine; T/Bilirubin: total bilirubin; AST: aspartate aminotransferase; ALT: alanine aminotransferase ; PT: prothrombin time; INR: international normalized ratio

Variable	Value	Units	Normal range
WBC	14.6	K/mm^3^	4.1 - 10.0
CO_2_	19	mmol/L	22 - 32
Crt	2.4	mg/dL	0.7 - 1.5
T/Bilirubin	3.9	mg/dL	0 - 1.5
AST	228	units/L	15 - 37
ALT	80	units/L	30 - 65
PT	20	seconds	9.8 - 13.9
INR	1.75	NA	0.9 - 1.15
Lactic acid	5.8	mmol/L	<2.0
Anion gap	15	mEq/L	4.0 - 12.0

**Figure 2:**
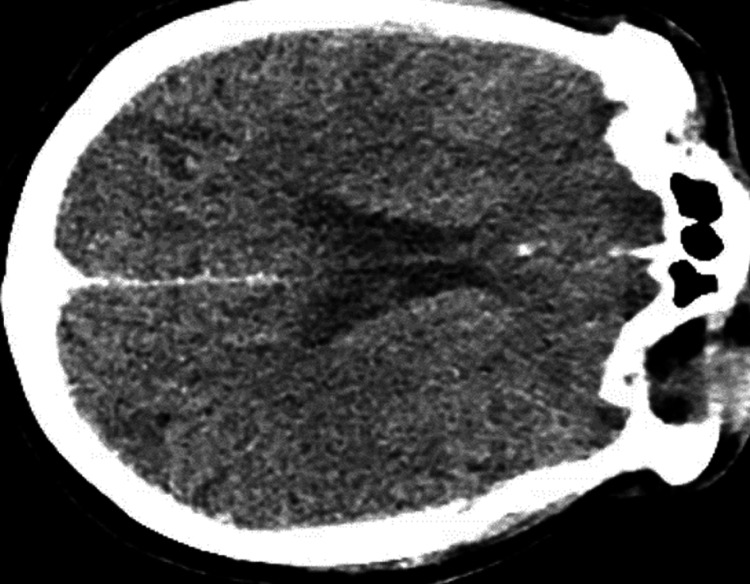
CT non-contrast of the head showing no evidence of an acute intracranial process CT: computed tomography

**Figure 3:**
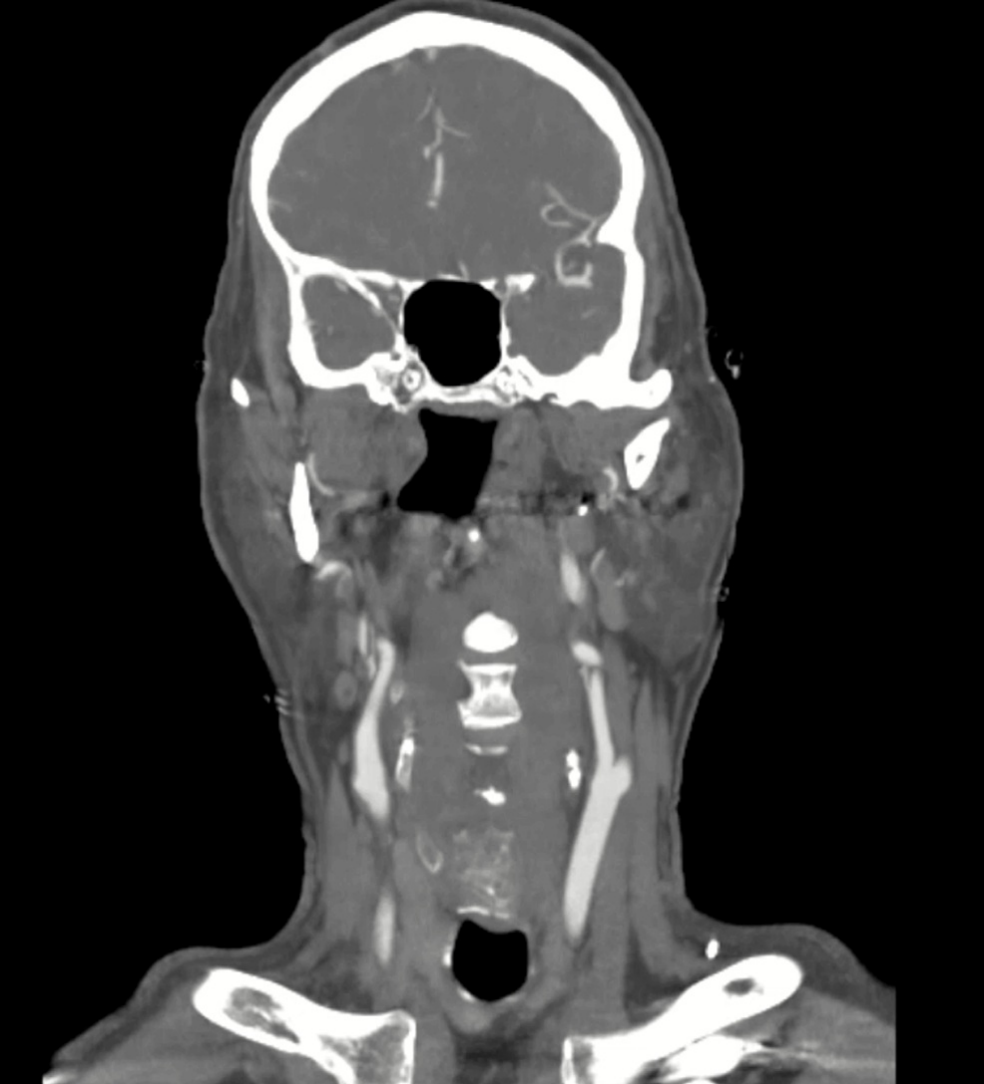
CT angiography of the head and neck revealing no significant large vessel occlusion or stenosis, or perfusion abnormalities CT: computed tomography

CT non-contrast of the head showed no evidence of an acute intracranial process (Figure 2). CT angiography of the head and neck was performed, revealing no significant large vessel occlusion or stenosis, or perfusion abnormalities (Figure 3).

Neurology was consulted, and they deemed the patient not to be a candidate for thrombolytic therapy due to the duration of the patient’s symptoms (three days). A sepsis protocol was initiated secondary to the fever, tachycardia, and potential source of infection (known colitis). Volume resuscitation, blood cultures, serial lactate measurements, and empiric broad-spectrum antibiotics were administered.

**Figure 4:**
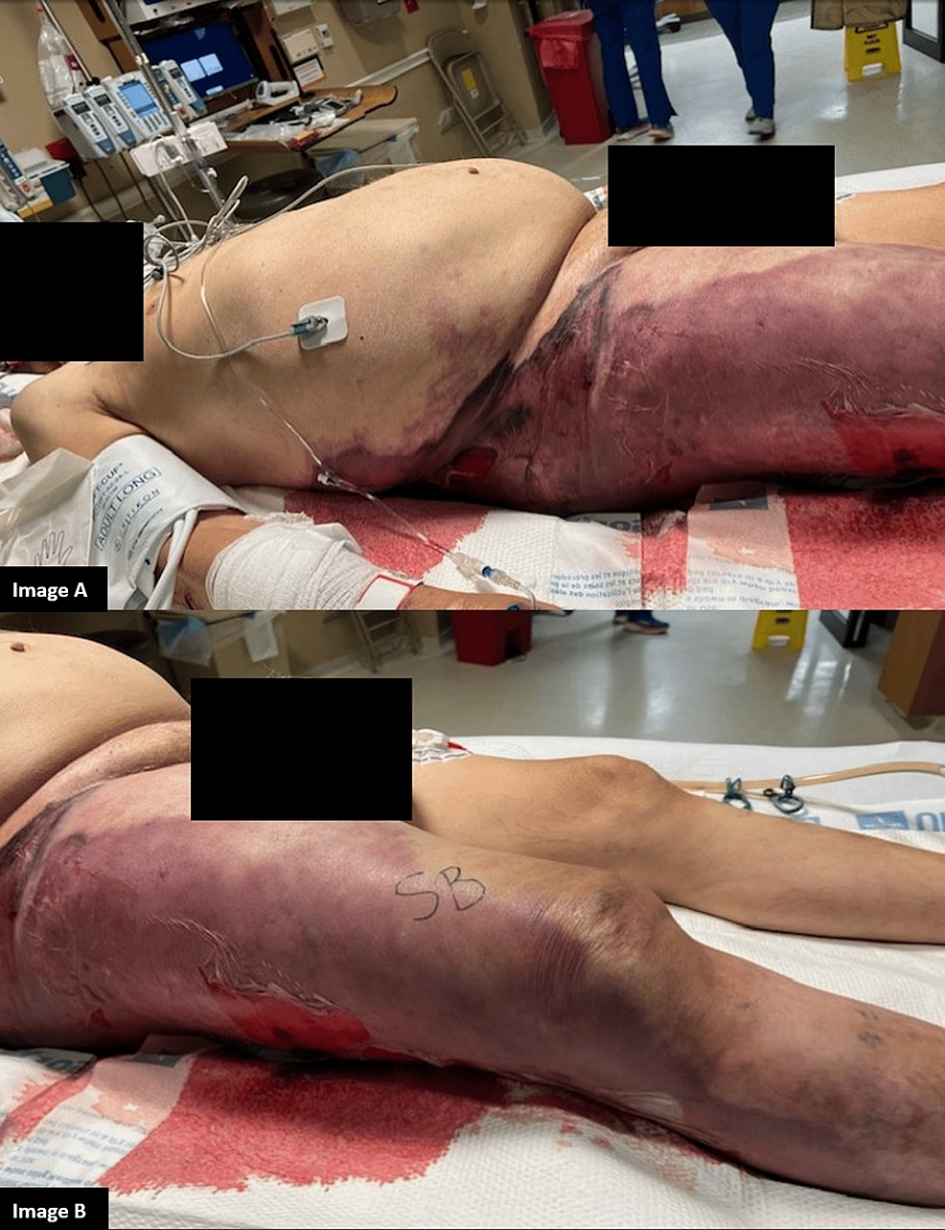
Macroscopic images showing the diffuse erythema with signs of NF extending from the right flank down the lateral aspect of the right lower extremity Image A shows the extension up to the right flank. Image B shows the infection extending to the lateral aspect of the right lower extremity NF: necrotizing fasciitis

Shortly thereafter, nursing noted significant erythema to the patient’s right lower extremity. On examination, hemorrhagic bullae and ecchymosis of the entire right lower extremity extending to the right flank were noted, raising concern for NF (Figure 4). A CT abdomen and pelvis and a CT of the right lower extremity were promptly ordered.

Before the patient was able to go to the CT scanner, a code blue was called. The patient was noted to have agonal breathing with an oxygen saturation of 50% and a blood pressure of 30s/20s mmHg. Advanced cardiac life support was initiated and return of spontaneous circulation (ROSC) was achieved. The patient received one push dose of epinephrine and was started on a Levophed drip. The patient was intubated and transferred to the ICU.

**Figure 5:**
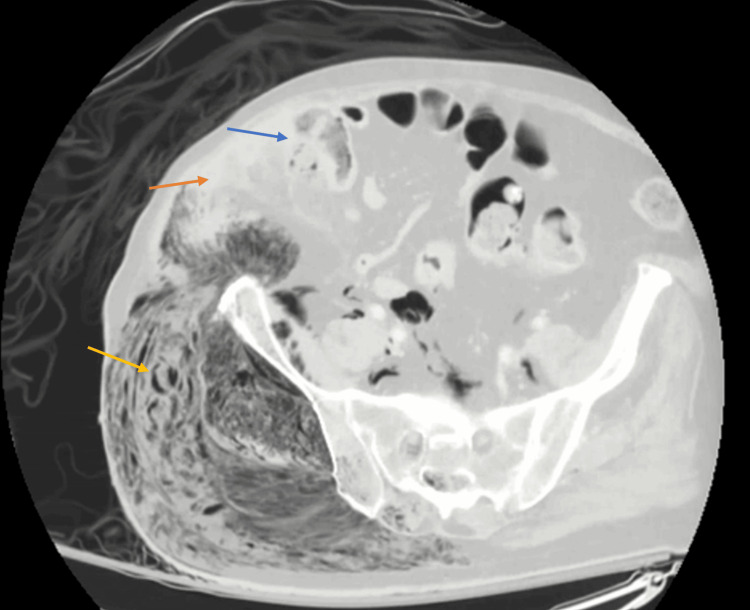
CT of the abdomen and pelvis revealing a cecal mass with micro-perforation The blue arrow shows the cecal mass and site of micro-perforation. The orange arrow shows the fluid tracking laterally out of the cecum. The yellow arrow shows the necrotizing infection and gas CT: computed tomography

**Figure 6:**
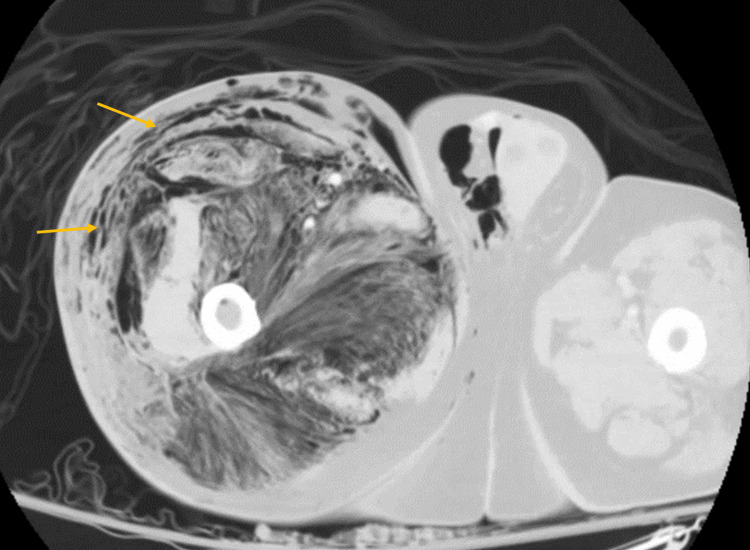
CT image revealing diffuse soft tissue gas present in the right lower extremity The yellow arrows show the diffuse gas present in the right lower extremity CT: computed tomography

CT imaging of the abdomen/pelvis and right lower extremity later showed a new-onset massive volume of gas in the soft tissues from the right flank through the right thigh and extending into the scrotum, within the pelvis, and the abdominal retroperitoneum (Figures 5-6). Findings suspicious of a cecal mass with micro-perforations were reconfirmed (Figure 5). Acute care surgery was consulted for emergency debridement of the necrotic tissue, but upon extensive discussion with the family, the patient was made Do Not Resuscitate (DNR) per his prior wishes. He expired early morning on hospital day two.

## Discussion

A prompt diagnosis of NF is crucial to limit morbidity and mortality associated with the condition. However, this is often difficult, as the condition is particularly rare and can often present with a paucity of clinical signs that might raise suspicion for a life-threatening disease process. Our patient initially presented with signs concerning for a cerebrovascular accident. Even though he met the sepsis criteria, a stroke workup took precedence. Additionally, the patient’s altered mental status, which was likely a manifestation of sepsis and organ failure, precluded an accurate, timely history, possibly delaying the diagnosis of NF. The lack of a detailed physical examination on presentation was also a factor.

NF can be classified into two types (it is to be noted that some authors consider NF secondary to Vibrio species a separate category). Type one is polymicrobial and involves non-group A streptococci with anaerobes. Type two involves group A β-hemolytic streptococci alone or in combination with a staphylococcus. The type of NF does not appear to affect clinical course or morbidity/mortality [4,5]. The organisms present in NF are typically dependent on the site of infection. Perineal and abdominal infections tend to be polymicrobial and grow enteric pathogens [5,6]. Extremity infections are more commonly monomicrobial and involve skin flora, especially Streptococcus pyogenes [5,6]. Considering the cecal perforation in our patient, our case most likely falls into the type one category.

While CT imaging from our patient’s initial visit to an outside emergency department the day before the presentation showed the cecal mass and perforation, it did not mention signs of NF, though early imaging is commonly negative [7]. Instead, the most common presenting signs are swelling, pain, and erythema [7]. It is unknown as to when the first signs of skin changes appeared in this patient, as it was not commented on in prior documentation and neither the patient nor his wife mentioned anything in that regard. It should be noted that frank gangrenous changes typically occur around day four or five of NF infection [5], suggesting that our patient might have had subtle skin changes that were mistaken for skin changes related to age, or just altogether missed. By the time any skin manifestations were noted, the patient had deteriorated considerably. The appearance of hemorrhagic bullae and gas gangrene are late signs, and septic shock at this stage has an extremely poor prognosis [2].

Moreover, laboratory findings of platelet <100,000 ul, abnormal coagulation studies, and elevated liver enzyme levels all relate significantly to the severity of NF, and our patient had all of these findings on presentation [8]. The Laboratory Risk Indicator for Necrotizing Fasciitis (LRINEC) score can be used as a diagnostic aid for NF (Table 2). This system measures C-reactive protein (CRP), white blood cell (WBC) count, hemoglobin, sodium, creatinine, and glucose and assigns each value a cumulative point. Less than or equal to 5 points suggests a low probability for the presence of NF; 6-7 points suggest a medium probability of NF; and 8 or more points suggest a high probability of NF. While this system is mainly used as a diagnostic tool, studies have shown that higher point totals (specifically above 7) can be an independent prognostic marker for disease lethality [9]. Our patient did not have a CRP level drawn, but his LRINEC score without the CRP was 5 points (WBC: 14.6 K/mm^3^, hemoglobin: 15.2 g/dL, sodium: 130 mmol/L, creatinine: 2.4 mg/dL, and glucose: 383 mg/dL). (It should be noted that there is also a modified LRINEC score that replaces sodium and glucose with erythrocyte count and fibrinogen levels).

**Table 2:** LRINEC scoring system LRINEC: Laboratory Risk Indicator for Necrotizing Fasciitis

Variables	Value	Score	Patient value	Normal range
C-reactive protein (mg/L)	≤150	0	Not Drawn	0.0 - 0.3
	>150	4		
Total white blood cell count (K/mm^3^)	<15	0	14.6	4.1 - 10.0
	15 - 25	1		
	>25	2		
Hemoglobin (g/dL)	>13.5	0	15.2	13.8 - 17.2
	11 - 13.5	1		
	<11	2		
Sodium (mmol/L)	≥135	0	130	137 - 145
	<135	2		
Creatinine (mg/dL)	≤1.6	0	2.4	0.7 - 1.5
	>1.6	2		
Glucose (mg/dL)	≤180	0	383	74 - 106
	>180	1		

The presence of diabetes mellitus has been found to negatively affect clinical outcomes, especially in the setting of high serum glucose levels on admission. Our patient had a diagnosis of type II diabetes mellitus, and his initial glucose level was 383 mg/dL. Diabetic patients have been shown to have longer lengths of stay, higher rates of complications, and higher morbidity and mortality compared to non-diabetic patients [3,10,11].

Another interesting component of this case is that the CT imaging report from the outside emergency department showed a perforated cecal mass, but the patient was sent home on antibiotics. A cecal mass predisposed this patient to cecal perforation and, had this been addressed before his second hospital presentation, he might have had a surgical intervention before he decompensated. His initial visit to the outside emergency department might have been the initial presentation of perforation of his cecum. NF of the retroperitoneum, abdominal wall, and perineum have all been reported to result from intestinal perforation/rupture [12-14]. Also, there is a possibility that this was a cancerous growth. The patient did have a history of prostate cancer. Of course, cancer leads to an immunocompromised state that would have made this patient more susceptible to NF infection. However, because this patient did not end up going to the operating room, biopsies of the cecal mass were not acquired, and hence its etiology is unclear.

Broad-spectrum antibiotic therapy should be initiated early in the disease process, but definitive treatment should involve surgical debridement [15]. Delays in surgical intervention significantly increase mortality [7], and studies have shown that the time of the first debridement is directly related to patient prognosis [7,16,17]. As mentioned earlier, the onset of NF in our patient remains unclear, with the need for surgical debridement only becoming apparent several hours after his presentation to the emergency department.

## Conclusions

This case report highlights the challenges in diagnosing NF, particularly when its presentation mimics other critical conditions such as stroke. The patient's initial presentation of altered mental status and stroke-like symptoms diverted attention from the underlying infectious process, resulting in a delay in diagnosis and intervention. Prompt recognition, early intervention, thorough physical examination, and a multidisciplinary management approach are crucial in mitigating the morbidity and mortality associated with NF. Continued education and awareness among healthcare providers are critical to improving outcomes and preventing delays in the diagnosis and treatment of NF.

## References

[1] Hua J, Friedlander P (2023). Cervical necrotizing fasciitis, diagnosis and treatment of a rare life-threatening infection. Ear Nose Throat J.

[2] Diab J, Bannan A, Pollitt T (2020). Necrotising fasciitis. BMJ.

[3] Chou PY, Hsieh YH, Lin CH (2020). Necrotizing fasciitis of the entire head and neck: literature review and case report. Biomed J.

[4] Giuliano A, Lewis F Jr, Hadley K, Blaisdell FW (1977). Bacteriology of necrotizing fasciitis. Am J Surg.

[5] Green RJ, Dafoe DC, Raffin TA (1996). Necrotizing fasciitis. Chest.

[6] McHenry CR, Piotrowski JJ, Petrinic D, Malangoni MA (1995). Determinants of mortality for necrotizing soft-tissue infections. Ann Surg.

[7] Meng Z, Wang Y, Chao J (2022). Extensive necrotizing fasciitis of scrotum and abdominal wall: report of two cases and a review of the literature. Front Surg.

[8] Teijido J, Damaschke A (2023). Case report: necrotizing fasciitis originating on the perineum and traversing to the anterior abdominal wall. Vis J Emerg Med.

[9] Hoesl V, Kempa S, Prantl L, Ochsenbauer K, Hoesl J, Kehrer A, Bosselmann T (2022). The LRINEC score-an indicator for the course and prognosis of necrotizing fasciitis?. J Clin Med.

[10] Zheng L, Yang C, Zhang W (2012). Is there association between severe multispace infections of the oral maxillofacial region and diabetes mellitus?. J Oral Maxillofac Surg.

[11] Cheng NC, Tai HC, Chang SC, Chang CH, Lai HS (2015). Necrotizing fasciitis in patients with diabetes mellitus: clinical characteristics and risk factors for mortality. BMC Infect Dis.

[12] Heidelberg LS, Pettke EN, Wagner T, Angotti L (2020). An atypical case of necrotizing fasciitis secondary to perforated cecal cancer. J Surg Case Rep.

[13] Bahl N, Long AS, Vemuri A, Jessee T (2021). A case of necrotizing soft tissue infection secondary to perforated colon cancer. Cureus.

[14] Marron CD, McArdle GT, Rao M, Sinclair S, Moorehead J (2006). Perforated carcinoma of the caecum presenting as necrotising fasciitis of the abdominal wall, the key to early diagnosis and management. BMC Surg.

[15] Falconi S, Wilhelm C, Loewen J, Soliman B (2023). Necrotizing fasciitis of the abdominal wall secondary to complicated appendicitis: a case report. Cureus.

[16] Edlich RF, Cross CL, Dahlstrom JJ, Long WB 3rd (2010). Modern concepts of the diagnosis and treatment of necrotizing fasciitis. J Emerg Med.

[17] Liu YM, Chi CY, Ho MW (2005). Microbiology and factors affecting mortality in necrotizing fasciitis. J Microbiol Immunol Infect.

